# The mystery of the fourth beat

**DOI:** 10.1007/s12471-015-0790-6

**Published:** 2016-01-04

**Authors:** F. van den Brink, I. Frenaij, R. van Tooren

**Affiliations:** St Antonius Hospital, Koekoekslaan 1, 3435 CM Nieuwegein, The Netherlands

**Fig. 1:**
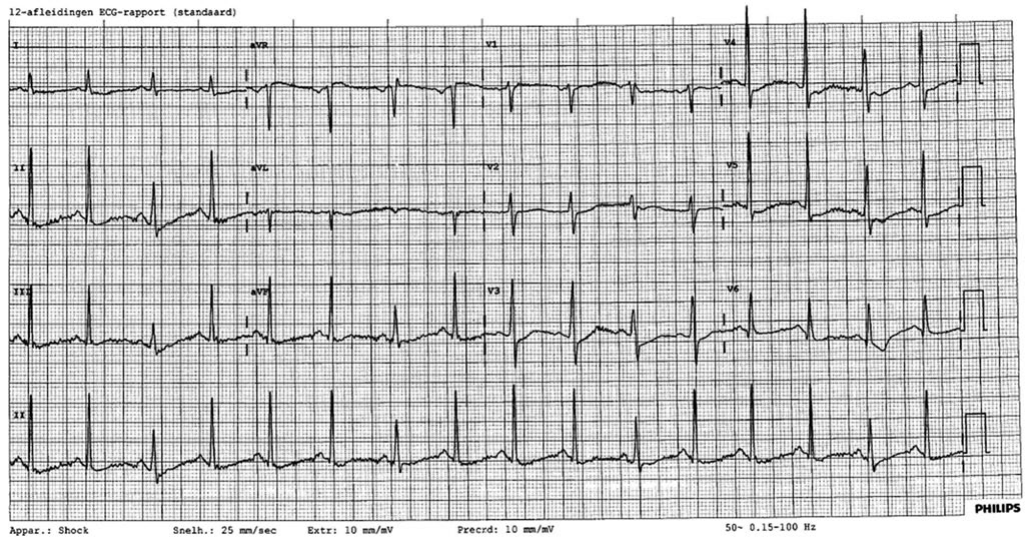
ECG upon admission

A previously fit and well 37-year-old woman collapsed on her doorstep. Her next door neighbour started cardiopulmonary resuscitation (CPR). A police car arrived at the scene as first responder. An automated external defibrillator was connected and administered two shocks in between which CPR was continued by the police officers. When the ambulance arrived she had a return of spontaneous circulation. Due to massive aspiration she was not intubated. Upon arrival at the Accident and Emergencies Department, we saw this ECG (Fig. 1). After stabilising the patient and intubating her, the ECG normalised almost completely.

What do you see?

Answer

You will find the answer elsewhere in this issue.

